# Effect of Eight-Week Transcranial Direct-Current Stimulation Combined with Lat Pull-Down Resistance Training on Improving Pull-Up Performance for Male College Students

**DOI:** 10.3390/life15010128

**Published:** 2025-01-20

**Authors:** Qian Li, Jiaqi Yan, Hanya Dai, Minjie Qiao, Mingxin Gong, Wenxin Niu, Ye Yang, Lejun Wang

**Affiliations:** 1 Sport and Health Research Center, Shanghai YangZhi Rehabilitation Hospital (Shanghai Sunshine Rehabilitation Center), Physical Education Department, Tongji University, Shanghai 200092, China; liqian234@126.com (Q.L.); debbiedaio@163.com (H.D.); 13611633515@163.com (M.G.); 2 School of Mathematical Sciences, Inner Mongolia University, Hohhot 010021, China; shadowof1128@163.com; 3 School of Physical Education, Shaanxi Normal University, Xi’an 710062, China; minjie0122@163.com; 4 School of Medicine, Tongji University, Shanghai 200092, China; niu@tongji.edu.cn; 5 School of Humanities, Tongji University, Shanghai 200092, China; 6 College of Physical Education and Health Science, Yibin University, Yibin 644000, China

**Keywords:** tDCS, resistance training, pull-up, biomechanics

## Abstract

The aim of this study was to investigate the effects and potential mechanisms of 8-week transcranial direct-current stimulation (tDCS) combined with resistance training (RT) on pull-up performance in male college students. Twenty-five male college students were randomly assigned to either RT combined with anodal tDCS stimulation (RT + tDCS) or RT alone (RT). Participants of both groups engaged in lat pull-down training programs for 8 weeks, with the RT + tDCS group receiving 20 min tDCS before each RT session. Pre- and post-intervention tests included pull-up endurance (number of repetitions), flexed arm circumference (FAC), and lat pull-down maximal voluntary isometric contraction (MVIC) peak force. During the pull-up endurance test, surface electromyography (sEMG) was recorded for the bicep brachii (BB), tricep brachii (TB), brachioradialis (BR), anterior deltoid (AD), middle deltoid (MD), posterior deltoid (PD), pectoralis major (PM), and latissimus dorsi (LD) muscles. Both groups demonstrated significant improvements in pull-up endurance and lat pull-down MVIC peak force after training, but no significant difference between the two groups was observed in the post-training test. Additionally, muscle activation of BR, PD, and PM decreased significantly in both groups, while the RT + tDCS group also demonstrated a significant reduction in TB coactivation after training. These findings suggest that eight weeks of tDCS combined with lat pull-down training and lat pull-down training alone can both significantly improve pull-up performance, which may be attributed to enhanced muscle contraction capacity. Although no significant training gains were found between the two training groups, the RT + tDCS group showed a significant decrease in TB coactivation and the enhancement of elbow flexion muscle contraction efficiency after training.

## 1. Introduction

A pull-up is a multi-joint exercise that effectively promotes muscular strength and endurance in the upper limbs and back. The exercise is an integral part of various physical activities such as climbing, rowing, swimming, and throwing [1,2], where strong pulling and pushing forces are required. Pull-up endurance tests are also included in some fitness assessments as a key indicator of strength and endurance, such as the National Physical Fitness Test and military fitness assessments in many countries [3,4]. However, a pull-up is inherently challenging because it requires considerable upper-body strength to lift one’s own body weight. Many people find it difficult to perform even a single pull-up and often struggle to increase their pull-up repetitions [5]. Therefore, improving pull-up endurance is essential for those aiming to enhance their overall physical fitness and performance in related activities.

RT is a widely recognized method for enhancing muscle strength [6], hypertrophy [7], and endurance [8] across various populations. Moreover, studies consistently demonstrate that RT effectively promotes neuromuscular adaptations [9], with its effects varying depending on the specific RT modality employed [6]. Among the commonly used RT exercises, lat pull-downs are notable for their effectiveness in recruiting similar back and upper arm muscles involved in pull-up execution [10]. There have been many studies that have considered the lat pull-down as a method to improve pull-up performance [5,11,12].

Transcranial direct-current stimulation (tDCS), as an emerging neuromodulation technology, has garnered growing research interest in the field of sports and exercise science. The tDCS is thought to modulate the excitability of neurons, thereby facilitating neural function and improving athletic performance [13]. It has been proposed that applying tDCS prior to training optimizes motor training-induced plasticity, leading to enhanced behavioral adaptations. Previous studies have demonstrated that anodal tDCS (a-tDCS) plays an important role in enhancing muscle strength [14], endurance [15], coordination [16], countermovement jump [17], and explosive force [18] by modulating cortical excitability in the primary motor cortex (M1) [19]. This suggests its potential to improve pull-up performance. However, some studies have found that a-tDCS does not offer additional benefits in significantly enhancing muscle strength and endurance [20]. The effects of a-tDCS in improving physical performance remain debated, and there is currently no research specifically examining its impact on pull-up performance. Thus, further investigation is needed to determine the potential benefits of a-tDCS in improving pull-up performance. There is also some research that has detected the effects of a combination of resistance training with a-tDCS. Hendy and Kidgell found that after 3 weeks of physical training, the a-tDCS group had significantly higher 5RM pull-up weight than the sham-tDCS group [21]. Conversely, another study reported no significant difference in 5RM pull-up performance between the a-tDCS and sham-tDCS groups after 6 weeks of combined tDCS and physical training for swimmers [22]. It remains to be determined whether a combination of a-tDCS and resistance training is effective in improving pull-up performance.

Therefore, the purpose of this study was to investigate the effects of a-tDCS combined with lat pull-down RT on pull-up performance in healthy male college students. Based on previous studies, it was hypothesized that the combined approach would improve pull-up performance more effectively than traditional lat pull-down RT alone.

## 2. Materials and Methods

### 2.1. Methodological Design of This Study

**Figure 1:**
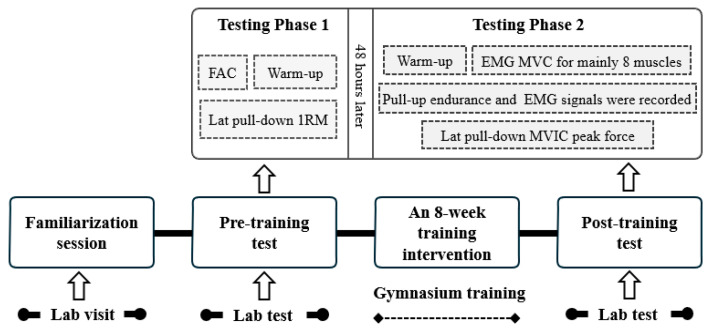
Methodological design of this study.

As shown in Figure 1, the procedure included familiarization, training intervention, and pre- and post-training assessments. Initially, participants underwent a one-week familiarization session to adapt to the laboratory environment and protocols. During this period, they practiced the lat pull-down RT in both conditions (experiencing and not experiencing the a-tDCS stimulation) to meet the experimental requirements. Baseline measurements were then taken. The procedure included two distinct phases, each separated by 48 h. In the first phase, flexed arm circumference (FAC) was conducted, followed by a warm-up and 1RM lat pull-down test. In the second phase, after the warm-up session, participants completed an electromyography (EMG) MVC test for normalization. Pull-up endurance was assessed with surface EMG signals being recorded during the exercise, and lat pull-down maximal voluntary isometric contraction (MVIC) peak force data were then collected. A 5 min rest was provided between each testing session. Participants were advised to avoid acute exercise, alcohol, and caffeine for 48 h before the measurements.

The 8-week training intervention consisted of two groups: the experimental group performed lat pull-down RT combined with a-tDCS intervention (RT + tDCS group), while the control group only performed the lat pull-down RT (RT group). The training was conducted in a gym setting. After the intervention, participants underwent post-training assessments, which involved repeating the pre-training measurements to evaluate the program’s effects.

### 2.2. Subject

Based on a power analysis using G*Power 3.1 software (Heinrich Heine, Dusseldorf, Germany) (effect size = 0.5, significance level = 0.05, statistical power = 0.80), 12 participants per group were needed for the repeated measures ANOVA. Consequently, 26 male college students were recruited and randomly assigned to the RT + tDCS group (n = 13) and RT group (n = 13). Ultimately, 25 participants completed the training, with one dropout in the RT + tDCS group due to personal reasons.

To be included in this study, participants had to be male college students who could perform at least one standard pull-up and were healthy, well-proportioned, and right-handed. They also needed to maintain a habit of physical training 2–3 times per week. Participants were excluded if they had a history of neuromuscular disorders, had recent head or upper limb injuries or surgeries, or had been involved in other studies with noninvasive electrical stimulation or upper limb strength training in the last 6 months. All participants were informed of the study protocol and provided written informed consent. This study was approved by the Tongji University Ethical Advisory Committee. Table 1 presents the basic characteristics of the participants.

### 2.3. Training Interventions

#### 2.3.1. RT Group Protocol

Participants in RT groups only completed lat pull-down exercises on the traditional lat pull-down strength trainer (Dr. Iron, Dr. Iron Fitness Equipment Company, Jiangsu, China) over an 8-week period (12 reps/set, 4 sets/session, 3 sessions/wk, with a total of 24 sessions).

Before each training session, a warm-up of approximately 5 min was performed, consisting mainly of upper body and back exercises. In the lat pull-down exercise, participants adjusted the seat height so that arms were fully extended and quadriceps were supported to begin the lift. Using a pronated grip slightly wider than shoulder width, participants completed each repetition. It took 6 s to complete every lat pull-down repetition, involving a rapid pull-down phase in the first 2 s, a sustained contraction phase in the third second, and a slow return phase in the last 3 s.

Following recommendations from previous studies [23,24,25] on moderate training volumes for optimal strength gains (60% to 80% of 1RM), the initial load was set at 65% of 1RM, with progressive increases of 5% every two weeks, reaching 80% of 1RM in the last 2 weeks of the training period.

#### 2.3.2. RT + tDCS Group Protocol

Prior to each resistance training session, participants in the RT + tDCS group were required to receive 20 min of tDCS stimulation. They wore a Halo Sport 2 headset (Halo Sports 2, Halo Neuroscience, San Francisco, CA, USA) to stimulate the primary motor area of the brain at an intensity of 2.2 mA. Before wearing the headset, the rubber pads of the headset needed to be fully moistened with water, and the moist foam electrodes covered the motor cortex area defined by the 10–20 internationally standardized lead system. Stimulation continued only when the headset maintained full contact with the scalp. Participants were required to wear the headset during stimulation and were advised to report any discomfort, with the option to withdraw from the study.

After stimulation, participants in the RT + tDCS group performed the same lat pull-down training program as those in the RT group above.

### 2.4. Procedures

#### 2.4.1. Pull-Up Endurance (Number of Repetitions)

Participants performed pull-ups starting from a dead hang position with their arms fully extended and locked and their feet off the floor. They grasped the bar with a pronated grip, with hands set wider than shoulder-width. From this starting position, participants lifted their bodies until their chins were above the bar. During the descent, they kept their bodies straight, lowering themselves in a controlled manner until their arms were fully extended again, without swinging. This procedure was repeated until participants could no longer complete a pull-up, and the total number of pull-ups was recorded. To ensure consistency and standardize the movement, the researcher observed and regulated the tempo throughout the exercise for all participants.

#### 2.4.2. Flexed Arm Circumference

Participants relaxed their arms, allowing them to hang naturally by their sides without muscle tension. They were then instructed to position their arm at a 90-degree angle with the elbow flexed and the bicep fully contracted. The largest circumference was measured using a flexible measuring tape and the result was recorded.

#### 2.4.3. One Repetition Maximum of Lat Pull-Down

The test was conducted following the guidelines of the American College of Sports Medicine [26]. The procedure started with a 5 min general warm-up. Participants then performed a warm-up set of 8 lat pull-down repetitions at 30% of their estimated 1RM to familiarize themselves with the lat pull-down exercise.

In the initial trial, participants completed lat pull-down repetitions at approximately 50–70% of their estimated 1RM, followed by a 1–3 min rest. After a 3 min rest, the load was increased by 5–10% of the initial load, ideally allowing participants to complete fewer repetitions. If successful, the weight was gradually increased until participants could complete only one repetition. If unsuccessful, the weight was reduced by 2.5–5% and the trial was repeated until only one repetition could be performed; 3 min of rest was provided between each trial. The highest successful load was recorded as the 1RM. Ideally, participants could reach their 1RM within four attempts.

#### 2.4.4. Surface Electromyography (sEMG)

In accordance with previously published reports and SENIAM recommendations, *electromyography* electrodes were placed collar-to-collar (2 cm) on the midbellies of the bicep brachii (BB), tricep brachii (TB), brachioradialis (BR), anterior deltoid (AD), middle deltoid (MD), posterior deltoid (PD), pectoralis major (PM), and latissimus dorsi (LD) muscles on the right side of the body. Before the electrodes were affixed, the skin was shaved with a razor, abraded with scrub, and carefully cleaned with a 75% alcohol cotton ball. EMG signals were collected by a wireless EMG system (BTS FREEEMG 1000, BTS, Garbagnate Milanese, MI, Italy) at a sample rate of 1000 Hz. MVC data for eight muscles (BB, TB, BR, PM, LD, AD, MD, PD) were measured with reference to the ABC of EMG and used for normalization.

#### 2.4.5. Lat Pull-Down Maximal Voluntary Isometric Contraction Peak (MVIC) Peak Force

Lat pull-down MVIC peak force was assessed by a homemade system consisting of two horizontal bars, a dumbbell bench, and a pull dynamometer (K-Pull, Kinvent, Montpellier, France). One of two bars was fixed to the wall, the upper end of the dynamometer was fixed under the bar, and the other end was connected to the bar of the lat pull-down machine via an iron chain.

To begin the test, participants were seated with their buttocks and thighs secured by straps for stability. They grasped the bar with a pronated grip at a distance of a little wider than their shoulder width. The seat height was adjusted so that the angles of shoulder and elbow flexion were 90° and 120°, respectively. Participants were instructed to exert maximal isometric force, as if performing a lat pull-down, and sustain this effort for 5 s. Every participant performed two trials, separated by a 3 s rest period. To promote maximal effort, verbal encouragement was provided during the trials. The pull dynamometer recorded the peak force at a sampling rate of 125 Hz. The highest force recorded from the two attempts, as determined by the device’s software, was used as the final result.

### 2.5. Data Processing

#### 2.5.1. Electromyography Activation Calculation

With Matlab R2019a (Mathworks, Natick, MA, USA), the acquired MVC data and pull-up sEMG signals for 8 muscles (BB, TB, BR, PM, LD, AD, MD, PD) were processed. Fourth-order Butterworth processed band-pass filtering of the sEMG signals from 5–500 Hz and full-wave rectification were performed. The moving average amplitude of the EMG signals was calculated and MVC was used as a standardized reference value during the pull-up EMG test.

The envelope of the sEMG was obtained by shifting the preprocessed sEMG signal, with a time window width of 50 ms to calculate the root mean squared (*RMS*) value. Calculating throughout the pull-up process, the average *RMS* amplitude was normalized to the *RMS* maximum:

(1) $$RMS=\frac{sqrt(\sum_{i=1}^{n}EMG_{i}^{2})}{N},$$

where *RMS* represents the calculated root mean square amplitude of the muscle and $EMG_{i}^{2}$ means the square of the muscle electromyographic amplitude at the *i*-th sampling point. *N* denotes the data length used for calculating *RMS*.

(2) $$RMS_{Normalized}=\frac{RMS_{raw}}{RMS_{MVC}},$$

where *RMS_Normalized_* and *RMS_raw_*, respectively, refer to the normalized and raw root mean square amplitudes of surface electromyographic signals collected during pull-ups and *RMS_MVC_* denotes the standardized reference value computed from sEMG signals recorded during *MVC* tests of the muscles.

#### 2.5.2. Electromyography Phase Synchronization Analysis

EMG signals were selected over the period in which the pull-up was performed. The selected EMG data were filtered for the frequency range 8–12 Hz (alpha band), 15–35 Hz (beta band), and 35–60 Hz (gamma band) by means of a 4th-order zero-phase-shift Butterworth filter. Phase synchronization index (*PSI*) in alpha, beta, and gamma frequency bands between EMG of synergistic (BB-BR, BB-PD, PM-LD, and PD-LD) and antagonist (TB-BB, TB-BR, AD-PD, and MD-LD) muscle pairs were calculated as follows:

(3) $$PSI=\sqrt{<cos\theta_{xy}^{H}\left(t\right){>}_{t}^{2}+<sin\theta_{xy}^{H}\left(t\right){>}_{t}^{2}},$$

where <.>*t* means the average of all the values.

(4) $$\theta_{xy}^{H}\left(t\right)=n\theta_{x}^{H}\left(t\right)-m\theta_{y}^{H}\left(t\right),$$

where *θH x*(*t*) and *θH y*(*t*) represent the phase angles calculated from the EMG signals of the antagonistic and synergistic muscle pairs using the Hilbert transform (with the muscle pairs matched as described above). In accordance with previous studies, the values of *m* and *n* were both assigned to 1 [27].

Data processing was performed using MATLAB R2019a software (Mathworks, Natick, MA, USA).

### 2.6. Statistical Analysis

Statistical analyses were performed using SPSS version 25.0 (SPSS, Inc., Chicago, IL, USA). All results were presented as the mean ± standard deviation (SD). Partial eta squared (η^2^) was reported for effect size, as 0.01 was considered small, 0.06 was considered medium, and 0.14 was considered large [28]. The normality and homogeneity of variances within the data were confirmed with the Shapiro–Wilk and Levene tests, respectively. Non-parametric tests were used for pull-up endurance. The Wilcoxon signed-rank test was used to compare pre- and post-intervention differences within each group, while the Mann–Whitney U test was used to assess differences in changes between the two groups during the pre- and post-test phases. For the other variables (lat pull-down MVIC peak force, EMG RMS, and PSI), two-way ANOVA [between factors: RT + tDCS group vs. RT group; within factors: pre- vs. post-training] repeated measures analysis of variance was used to assess differences between the participants. Statistical significance was set at *p* < 0.05.

## 3. Results

### 3.1. Pull-Up Endurance (Number of Repetitions)

**Figure 2:**
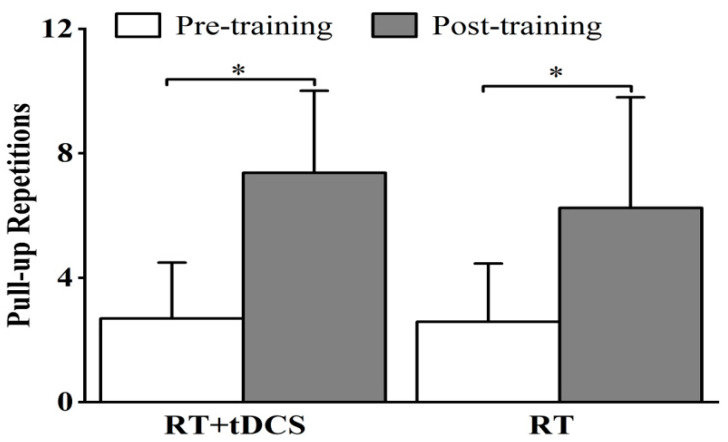
Pre- to post-training mean and SD of the maximal number of pull-up repetitions changes for the RT + tDCS and RT groups. * Significant pre- to post-training increases in the number of pull-up repetitions.

Figure 2 displays the pull-up endurance results for the participants in the RT + tDCS and RT groups measured before and after 8 weeks of training. After training, pull-up repetitions increased from 2.69 ± 1.80 and 2.58 ± 1.88 to 7.38 ± 2.63 and 6.25 ± 3.55 for the RT + tDCS and RT groups, respectively. Statistical analysis showed a significant increase in RT + tDCS (*p* = 0.001) and RT groups (*p* = 0.002) after training compared to pre-training. There was no significant difference between groups before (*p* = 0.755) or after (*p* = 0.157) training.

### 3.2. Flexed Arm Circumference

**Figure 3:**
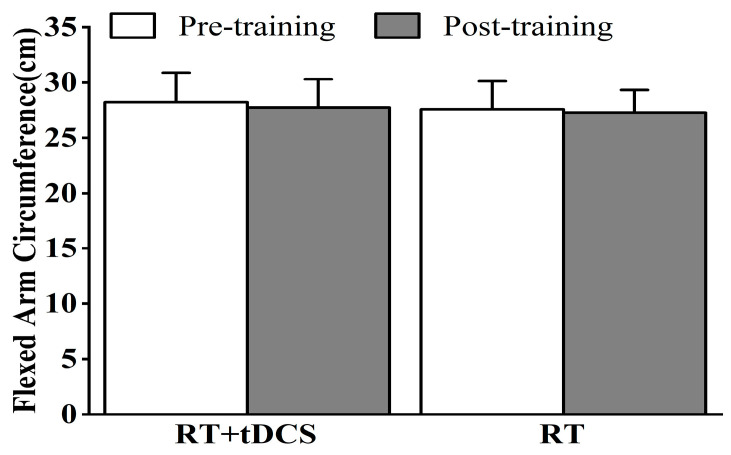
Pre- to post-training mean and SD of the flexed arm circumference for the RT + tDCS and RT groups. No significant difference was found.

The flexed arm circumference results for the RT + tDCS and RT groups before and after 8 weeks of training are presented in Figure 3. The FAC of the RT + tDCS group during pre- and post-training tests was 28.23 ± 2.66 and 27.75 ± 2.55 cm, whereas the values for the RT group were 27.59 ± 2.53 and 27.29 ± 2.02 cm. The findings revealed no significant main effect of training time (F = 2.61, *p* = 0.12, ES = 0.10) or group (F = 0.27, *p* = 0.61, ES = 0.01). Additionally, no interaction effect of training time and group was observed (F = 0.03, *p* = 0.87, ES < 0.01).

### 3.3. Lat Pull-Down Maximal Voluntary Isometric Contraction Peak Force

**Figure 4:**
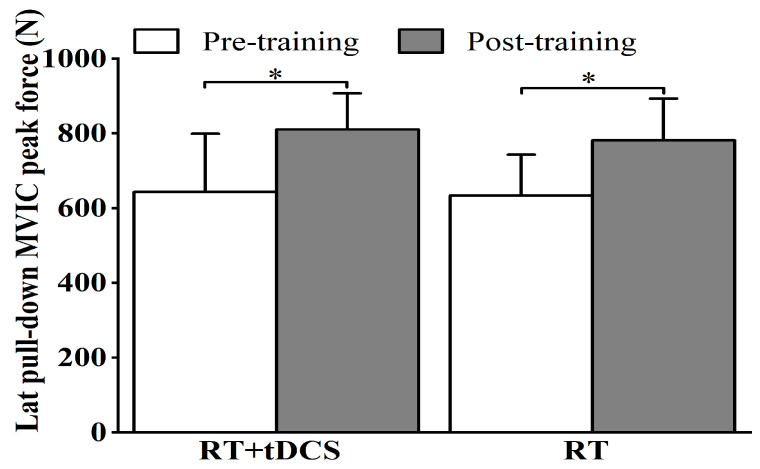
Pre- to post-training mean and SD of the lat pull-down maximal voluntary isometric contraction (MVIC) peak force for the RT + tDCS and RT groups. * Both groups showed stronger force following 8 weeks of training.

Figure 4 depicts the results for lat pull-down MVIC peak force for participants in the RT + tDCS and RT groups before and after 8 weeks of training. The repeated measures analysis revealed a significant main effect of time (F = 98.62, *p* < 0.01, ES = 0.81) on lat pull-down MVIC peak force. No significant main effect of group (F = 0.18, *p* = 0.68, ES = 0.01) nor interaction effect of time and group (F = 0.36, *p* = 0.55, ES = 0.02) was found. Participants in both groups showed an average of 24.7% increase in lat pull-down MVIC peak force during the post-training test compared to pre-training.

### 3.4. Electromyography Muscle Activation

#### 3.4.1. Agonist Muscle Activation

The results of EMG RMS amplitudes of agonist muscle activation for participants in the RT + tDCS and RT groups before and after 8 weeks of training are shown in Table 2. The data analysis indicated significant main effects of training time on BR (F = 17.79, *p* < 0.01, ES = 0.44), PM (F = 5.34, *p* = 0.03, ES = 0.19), and LD (F = 10.08, *p* = 0.004, ES = 0.04) muscles. No significant main effects of training time were found on BB (F = 3.17, *p* = 0.09, ES = 0.12) or PD (F = 3.08, *p* = 0.09, ES = 0.12). Additionally, no significant main effects of group or interaction effects of training time and group were observed for any of the five agonist muscles. For both groups, there was an average decrease of 30.6%, 13.8%, and 32.4% on BR, PM, and LD muscles, respectively, during the post-training test compared to pre-training. Participants in both groups also significantly reduced their EMG RMS amplitudes of BR muscles compared to their pre-training results.

#### 3.4.2. Antagonist Muscle Coactivation

Table 3 illustrates the EMG RMS amplitude results of antagonist muscle coactivation for participants in the RT + tDCS and RT groups before and after 8 weeks of training. The statistical data showed that although there were no significant main effects of time (F = 2.53, *p* = 0.13, ES = 0.10) or group (F = 1.72 *p* = 0.20, ES = 0.07) on TB muscle coactivation, a significant interaction effect of group and time was observed (F = 5.00, *p* = 0.04, ES = 0.18). This was primarily due to a significant 27% decrease in the RMS of TB muscle coactivation in the RT + tDCS group post-training compared to pre-training. No significant changes in AD and MD muscle coactivation were observed in either group.

### 3.5. Electromyography Phase Synchronization Index (PSI)

#### 3.5.1. Antagonistic Muscle Pairs

**Figure 5:**
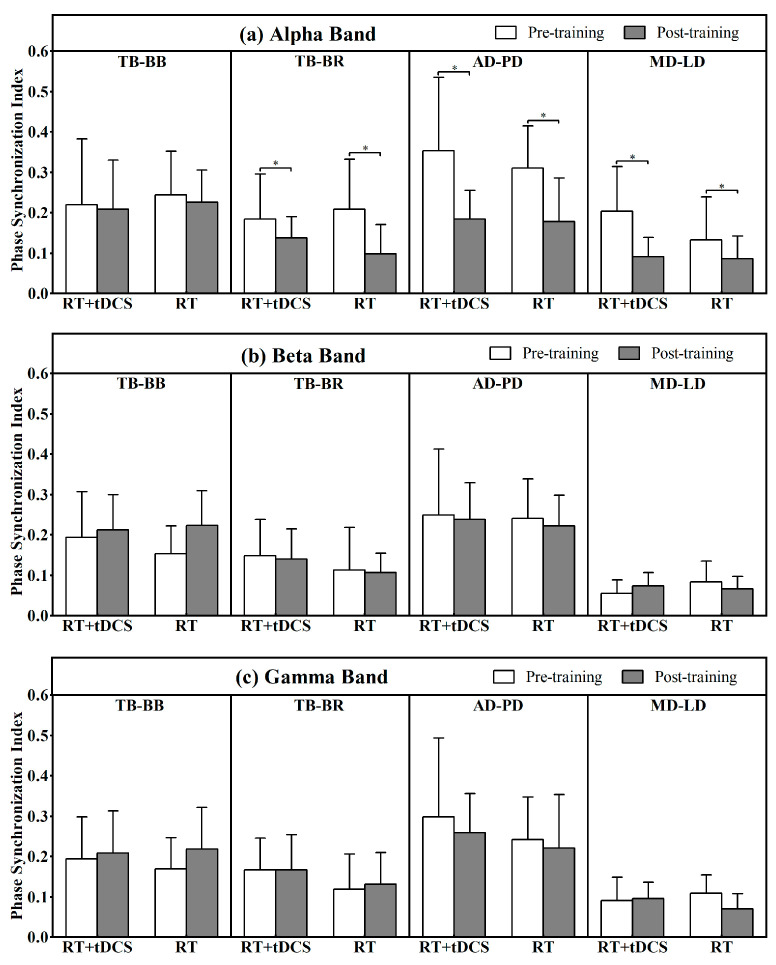
Changes in Phase Synchronization Index (PSI) for muscle pairs TB-BB (The first column), TB-BR (the second column), AD-PD (the third column), and MD-LD (the fourth column) for alpha (four subfigures of the upper row), beta (four subfigures of the middle row), and gamma (four subfigures of the bottom row) frequency bands for RT + tDCS and RT groups. Pre- to post-training mean and SD of the electromyography phase synchronization index (PSI) in antagonist muscle pairs changes for RT + tDCS and RT groups. * Significant difference in PSI before and after training.

Figure 5 reveals the results of the PSI of antagonistic muscle pairs during pull-up execution across alpha, beta, and gamma frequency bands before and after the 8-week training period. In the alpha band, significant main effects of training time were found on the TB-BR (F = 8.68, *p* = 0.007, ES = 0.27), AD-PD (F = 23.53, *p* < 0.01, ES = 0.06), and MD-LD (F = 11.36, *p* = 0.003, ES = 0.33) antagonistic muscle pairs, with no significant main effects of group. Specifically, participants in the RT + tDCS group showed a significant decrease in the PSI by 47.7% for the AD-PD pair and 55.8% for the MD-LD pair. Similarly, participants in the RT group exhibited a significant decrease in PSI by 52.7% for the TB-BR pair and 42.8% for the AD-PD pair. In the beta and gamma bands, no significant differences were observed for any of the four antagonistic muscle pairs.

#### 3.5.2. Synergistic Muscle Pairs

**Figure 6:**
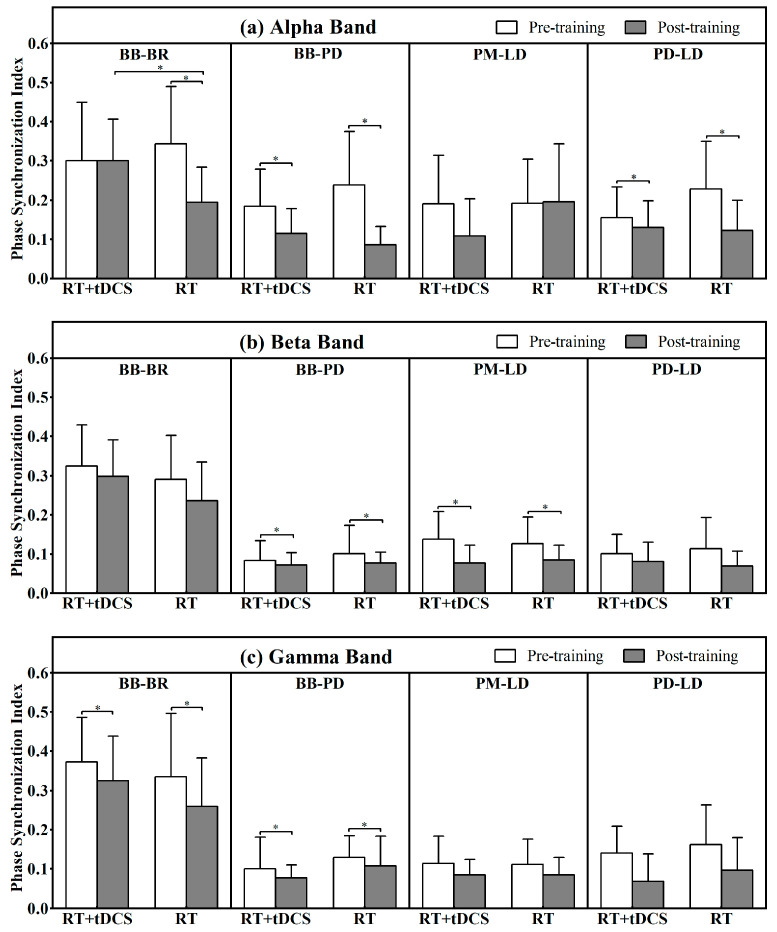
Changes of muscle pairs BB-BR (The first column), BB-PD (the second column), PM-LD (the third column), and PD-LD (the fourth column) for alpha (four subfigures of the upper row), beta (four subfigures of the middle row), and gamma (four subfigures of the bottom row) frequency bands for RT + tDCS and RT groups. Mean and SD of the electromyography phase synchronization index (PSI) in synergistic muscle pairs changes pre- to post-training for the RT + tDCS and RT groups. * Significant pre- to post- training increases in PSI.

Figure 6 depicts the results of PSI on the synergistic muscle pairs across alpha, beta, and gamma frequency bands before and after the 8-week training period.

For the BB-BR muscle pair, significant main effects of training time were detected in both the alpha (F = 5.48, *p* = 0.03, ES = 0.19) and gamma (F = 6.17, *p* = 0.02, ES = 0.21) bands. In the alpha band, a significant interaction effect of training time and group (F = 5.47, *p* = 0.03, ES = 0.19) was identified. After 8 weeks of training, participants in the RT group showed a significant PSI decrease of 43.3% in the alpha band and 22.7% in the gamma band. However, the post-training PSI of the RT + tDCS group was significantly higher than that of the RT group in the alpha band.

The results demonstrated a significant main effect of training time in the alpha band on the BB-PD muscle pair’s PSI (F = 10.95, *p* = 0.003, ES = 0.32), with participants in the RT group showing a significant reduction of 46.3% in PSI post-training compared to pre-training.

A significant main effect of training time on the PSI of the PM-LD muscle pair (F = 11.21, *p* = 0.003, ES = 0.33) was observed in the beta band, with a notable reduction in PSI in the RT + tDCS group post-training compared to pre-training.

For the PD-LD muscle pair, a main effect of training time was observed in the alpha band (F = 14.09, *p* = 0.001, ES = 0.38), with the RT group exhibiting a significant reduction of 64.2% in the post-training test compared to pre-training.

## 4. Discussion

This study aimed to investigate the effects of combining a-tDCS with resistance training on pull-up performance in healthy male college students. Improving pull-up performance is important not only for individuals enhancing their fitness but also for athletes requiring strong upper-body pulling strength. This study found that the maximal number of pull-up repetitions performed by participants in both the RT + tDCS and RT groups significantly increased after an 8-week training intervention. However, no significant difference was observed in pull-up endurance between the two groups during the post-training test compared to pre-training. Furthermore, after the training intervention, the agonist activation levels of the BR, PD, and PM muscles decreased significantly in both groups, whereas the RT + tDCS group also exhibited a significant reduction in the antagonist coactivation of the TB muscle.

The effect of resistance training can be influenced by many factors such as the specificity of the training tasks, the design of the training program, and individual differences. Regarding the effect of RT on improving pull-up endurance performance, significant improvements in training gains have been revealed by previous research. For example, Jones [29] found significant improvements in pull-up endurance performance after a 15-week off-season training program of progressive-overload whole-body vibration combined with RT. In a study including a 12-week strength and aerobic training program, the percentage of college-aged women participants who could perform a pull-up increased from 10.5% to 31.6% [30]. However, inconsistent findings have also been found in previous studies. For example, Vigouroux and Devise [31] observed no significant changes in pull-up endurance among elite climbers after 5-week eccentric, isometric, and plyometric pull-up training. In this study, after an 8-week lat pull-down resistance training intervention, the repetition number of pull-ups in the RT group increased from 2.58 ± 1.88 to 6.25 ± 3.55, demonstrating significant training gains after the 8-week lat pull-down resistance training.

Although the effect of tDCS on sports performance has been widely investigated in previous research, few studies have combined a-tDCS with resistance training to enhance endurance performance. Previous studies in healthy populations have consistently shown no significant improvements in endurance performance with this combination. For instance, Yang et al. [22] found no significant enhancement of self-weight pull-up endurance performance after 6 weeks of a-tDCS combined with physical training for elite swimmers. Similarly, Jung et al. [20] found that a-tDCS failed to demonstrate its effectiveness in enhancing endurance scores in healthy adults compared to the sham-tDCS group after 6 weeks of a-tDCS combined with physical training. Our study’s results align with these findings, as no significant differences were observed in pull-up endurance between the RT and RT + a-tDCS groups after training. The large effect size for time (ES = 0.84) also indicates a meaningful improvement in pull-up endurance, with a substantial proportion of variance explained by training time. This may be due to differences in stimulation protocols, as tDCS was not applied before the assessment [32], limiting its potential to improve pull-up performance. Additionally, the precision of tDCS targeting could influence outcomes, as different muscles and abilities correspond to distinct cortical areas [33].

Previous research suggested that short-term resistance training may not induce significant muscle hypertrophy, and short-term resistance training gains are mainly attributed to neural adaptations, including improvements in agonist muscle contractility and reductions in antagonist muscle coactivation levels [34,35]. In this study, there was no significant difference in flexed arm circumference between the pre- and post-training tests for both groups of participants, while both the RT + tDCS and RT groups showed a significant decrease in the activation levels of the BR, PM, and LD agonist muscles after training. This reduction indicates that participants could perform the exercises with less muscle activation. Since the resistance load of pull-ups did not change significantly during the pre-and post-training test, the decline in muscle activation may have been mainly due to the increase in the muscle capacity to induce maximum muscle strength, which was confirmed by the increase in MVIC peak force after training. However, there were no significant differences in the activation levels of agonist muscles between the tDCS + RT and RT groups before and after training. The results demonstrated that adding an a-tDCS intervention including lat pull-down resistance training may not bring additional training gains to muscle contraction capabilities.

Antagonist muscle coactivation levels are a critical indicator of central nervous system efficiency. Lower coactivation levels suggest that the central nervous system controls the muscles in a more efficient manner [36]. The reduction of coactivation is seen as a key mechanism in training-induced muscle strength gains and performance improvements [37]. However, ongoing debate about the impact of training on coactivation has been revealed in previous research. While some studies found that training significantly reduced coactivation levels [38], others suggested that increased muscle strength was not necessarily related to decreased coactivation [39]. In this study, participants in the RT + tDCS group exhibited a significant reduction in TB coactivation levels during the post-training test. The findings suggest that 8 weeks of RT combined with a-tDCS significantly reduces antagonist muscle coactivation during pull-up exercises. This effect may be attributed to the effect of tDCS to modulate cortical activity, optimizing the coordination of muscle coactivation by the central nervous system during training, which decreases antagonist muscle coactivation and improves motor skill performance [40]. However, no other significant changes in antagonist muscle coactivation were observed in either group compared to pre-training. This indicates that the current training protocols did not significantly affect coactivation levels in antagonist muscles except for TB in the RT + tDCS group.

It has been suggested that PSI between the EMG of co-contracted muscles in the alpha frequency band is related to Ia afferent feedback [41]. Intermuscular interactions in the beta frequency band have been suggested to be closely related to the common corticospinal drive from the motor cortex to the muscles in low and moderate isometric voluntary contractions [37], while PSI in the gamma frequency band may be related to the common neural inputs of the co-contracted muscles in strong isometric and dynamic voluntary contractions [42,43,44]. In the current study, significant decreases in PSI in the beta or gamma frequency band were found in synergistic muscle pairs of BB-BR, BB-PD, and PM-LD, while a significant decline in PSI was only found in the alpha band for antagonistic muscle pairs. The results may indicate that the training-induced neural adaptations observed in the current research were mainly achieved by the optimization of the coordinated control of synergistic muscles rather than antagonistic muscles, which is consistent with the results of the decline in agonist muscle activation rather than antagonist muscle coactivation discussed above.

It should be acknowledged that this study has several limitations. Firstly, considering the feasibility of the experiment and referring to relevant previous research, a sham-tDCS group was not included in the current research; thus, the potential psychological effects of tDCS on the participants cannot be fully excluded. Secondly, this study exclusively recruited male participants and did not include female subjects, which restricts the generalizability of the findings across genders. Thirdly, in the collection of sEMG signals, cross-talk is an inevitable concern. In this study, we employed the same trained researcher to position the electrodes in assessments, which may have minimized potential crosstalk effects. Finally, individual variations in familiarity and adaptation to the movement rhythm during the pull-ups may have affected the consistency of the test results.

The results of this study further highlight their significant practical application. The findings suggest that incorporating lat pull-down exercises into training regimens can effectively strengthen upper body muscles for recreationally active individuals. Moreover, integrating tDCS during training may optimize muscle contraction efficiency. The tDCS used in this study was administered via headphones, making it an accessible addition to training programs.

## 5. Conclusions

Eight weeks of a-tDCS combined with a lat pull-down RT program and a lat pull-down RT alone program could both effectively improve pull-up performance among male college students, which may have been due to the enhanced muscle contraction capacity. Although no significant training gains were found between the two training groups, the RT + tDCS group showed a significant decrease in TB coactivation and an enhancement of elbow flexion muscle contraction efficiency after training.

## Figures and Tables

**Table 1:** The basic characteristics of the participants.

Group	N	Age (yrs)	Height (cm)	Weight (kg)	BMI (kg/m^2^)
RT + tDCS	13	19.08 ± 1.26	175.07 ± 4.84	63.71 ± 8.38	20.75 ± 2.75
RT	12	18.33 ± 0.49	173.88 ± 7.09	61.23 ± 5.05	20.33 ± 2.07

**Table 2:** EMG RMS amplitudes (%MVC) of agonist muscle.

	RT + tDCS		RT	
	Pre-Testing	Post-Testing	Pre-Testing	Post-Testing
BB	0.72 ± 0.39	0.55 ± 0.19	0.60 ± 0.26	0.56 ± 0.09
BR	0.79 ± 0.18	0.56 ± 0.13 ^△^	0.90 ± 0.34	0.62 ± 0.12 ^△^
PM	0.32 ± 0.10	0.28 ± 0.13 ^ △^	0.33 ± 0.11	0.28 ± 0.10 ^△^
LD	0.39 ± 0.18	0.24 ± 0.07 ^ △^	0.42 ± 0.18	0.31 ± 0.13 ^△^
PD	0.41 ± 0.14	0.36 ± 0.10	0.43 ± 0.18	0.35 ± 0.09

Note: BB, BR, PM, LD, and PD denote bicep brachii, tricep brachii, pectoralis major, latissimus dorsi, and posterior deltoid fasciculus, respectively, and ^△^ denotes a significant difference in the level of muscle activation before and after training (*p* < 0.05).

**Table 3:** EMG RMS amplitudes (%MVC) of antagonist muscle.

	RT + tDCS		RT	
	Pre-Testing	Post-Testing	Pre-Testing	Post-Testing
TB	0.50 ± 0.22	0.37 ± 0.09 ^△^	0.36 ± 0.12	0.38 ± 0.15
AD	0.15 ± 0.17	0.10 ± 0.11	0.11 ± 0.08	0.09 ± 0.06
MD	0.22 ± 0.11	0.18 ± 0.06	0.20 ± 0.12	0.17 ± 0.05

Note: TB, AD, and MD denote tricep brachii, anterior deltoid, and middle deltoid muscles, respectively; ^△^ denotes a significant difference in the level of muscle activation before and after training (*p* < 0.05).

## Data Availability

The original contributions presented in this study are included in the article; further inquiries can be directed to the corresponding author.
